# Turbulent-like Dynamics Predict Treatment Outcome in Major Depressive Disorder

**DOI:** 10.21203/rs.3.rs-7208894/v1

**Published:** 2025-09-09

**Authors:** Henricus Ruhe, Maarten Poirot, Yonatan Sanz Perl, Matthan Caan, Henk Marquering, Gustavo Deco, Morten Kringelbach, Liesbeth Reneman

**Affiliations:** Radboudumc; Universitat Pompeu Fabra; Universitat Pompeu Fabra; University of Oxford; Amsterdam UMC - University of Amsterdam

**Keywords:** Major Depressive Disorder, Antidepressant Treatment Response, Resting State Functional Magnetic Resonance Imaging, Turbulence, Machine Learning

## Abstract

**INTRODUCTION:**

Major depressive disorder is a prevalent and debilitating disease. Finding effective treatment is a lengthy trial-and-error process. Thus, identifying predictors of treatment outcome is key to reducing disease burden. Brain turbulence-like dynamic measures on transmission of information might be a potential methodological strategy for identifying informative predictors of antidepressant treatment response.

**METHODS:**

We analyzed data of the EMBARC study in which 296 adult outpatients were randomized to eight weeks of sertraline or placebo treatment. Resting state functional MRI scans were acquired at baseline and one week after treatment initiation. Turbulence-like dynamic measures were computed. Generalized linear models were used to predict week 8 response and symptom severity scores. Primary performance metrics were the area under the receiver operating characteristic (AUROC) and the root mean squared error (RMSE) respectively. Permutation testing was performed to test for significance against chance. Internal leave-one-out cross-validation was performed on the sertraline-treated patients (sample A) and external validation on the placebo arm (sample B) and on placebo-treated, non-responding patients who later switched to sertraline (sample C).

**RESULTS:**

226 patients were analyzed (age 37.9±13.4 years; 148 [65.5%] female). Sample A included 109 participants, sample B 121, and sample C 61. Internal cross-validation results were significantly better than chance at predicting response (AUROC=0.71, balanced accuracy=69.5%, p=0.008) and symptom severity (RMSE=4.7, p=0.025). External validation in sample B did not yield performance significantly better than chance, and in sample C only prediction of response did (AUROC=0.65, balanced accuracy=60.3%, p=0.038).

**CONCLUSION:**

The turbulence framework is a suitable paradigm for predicting sertraline response in major depressive disorder. Prediction specificity to sertraline treatment was limited. Non-responders to sertraline were characterized by increased long-distance and reduced short-distance information cascade flow.

## 1. INTRODUCTION

Major Depressive Disorder (MDD) is a widespread chronic mental health condition, affecting one in five adults over the course of their lives (Lim et al., 2018). It is the leading cause of disability globally (Friedrich, 2017; Malhi & Mann, 2018). Given its profound impact on quality of life and the significant societal costs it incurs (Greenberg et al., 2021), finding effective treatments for MDD is crucial.

With 97 million daily doses in OECD countries, MDD is commonly treated with monoaminergic antidepressant medications (Brauer et al., 2021; OECD, 2022). However, with a wide range of available treatments, up to 50% of patients fail to respond to the first antidepressant, and remission rates are even lower (~30%) (Trivedi et al., 2006). As a result, many patients are subjected to multiple sequential treatments (Al-Harbi, 2012), prolonging their disease burden, increasing exposure to side effects, and driving up societal costs (Greenberg, Fournier, Sisitsky, Pike, & Kessler, 2015). With a modest average effect size of antidepressant efficacy compared to placebo (0.30) (Khan & Brown, 2015) pre-treatment tools to identify patients who will respond or not would be valuable to support personalized antidepressant treatment selection (Phillips et al., 2015). However, efforts to identify clinical, sociodemographic, or biological predictors of treatment response have so far had limited success (Kraus, Kadriu, Lanzenberger, Zarate Jr, & Kasper, 2020).

Potential predictors of treatment outcome have been identified in the brain at rest (Dichter, Gibbs, & Smoski, 2015; Mulders, van Eijndhoven, Schene, Beckmann, & Tendolkar, 2015). With functional magnetic resonance imaging (fMRI) functional connectivity patterns intrinsic to the brain can be measured at rest or during a task. Resting state fMRI (rs-fMRI) studies are considered to reflect more traits of response than task-based fMRI studies (Kang, 2015). Several large-scale resting-state networks (RSNs) have been identified in the rs-fMRI connectivity patterns. These networks, as well as the connectivity between these networks, have been found to be disturbed in MDD (Gudayol-Ferré, Peró-Cebollero, González-Garrido, & Guàrdia-Olmos, 2015). Meta-analyses have found two associations with antidepressant treatment outcome. I. increased functional connectivity between frontal and limbic brain regions is associated with better response rates, possibly resulting in greater inhibitory control over neural circuits that process positive and negative emotions, assign salience, and regulate cognitive and emotional control (Kaiser, Andrews-Hanna, Wager, & Pizzagalli, 2015; Mulders et al., 2015; Sheline et al., 2009; Zhu et al., 2012); II. hyperconnectivity within the default mode network (DMN) and hypoconnectivity of the cognitive control network. The DMN is hypothesized to support internally directed and self-referential thought. Thus, its hyperconnectivity provides a neural basis for the rumination often seen in depressive patients (Hamilton, Farmer, Fogelman, & Gotlib, 2015; Kaiser et al., 2015). Hypoconnectivity within the CCN may limit cognitive flexibility and hinder adaptive emotional regulation (Cole, 2014). These associations with antidepressant treatment outcome could play a critical role in the development of a treatment decision support tool.

Previous studies attempting treatment response using rs-fMRI have thus far failed to produce clinically meaningful predictive performance (Gerlach, 2022). Few consistent findings have emerged to provide a reliable basis for predicting antidepressant treatment response, but results were generally inconsistent (Dichter, 2013). Although many of these studies have been constrained by small sample sizes, even large-scale, multi-site studies have reported inconsistent hyper- and hypo-connectivity (Kaiser, 2015; Yan, 2019). A limitation that even these works suffer from is their analytical methods. Common approaches include regional homogeneity (Guo, 2012a; Guo, 2012b; Lai, 2012; Wang, 2014; Wu, 2011) seed-based analyses (Alexopoulos, 2012; Anand, 2007; Menze, 2015; Kozel, 2011; Lui, 2011; Ma, 2012; Posner, 2013; Yang, 2014; Guo, 2013a; Guo, 2013b), and independent component analysis (Li, 2013; Poirot, 2024; Abbott, 2013). A fundamental limitation of these methods is their inability to capture the dynamic, time-varying nature of brain connectivity. These static approaches reduce connectivity dynamics over time to a single snapshot, which may not fully represent the complexity of brain activity. Thus, there is a need for methods better suited to capturing the dynamic interactions within the brain.

Recent studies have shown that the way information is transferred through the brain follows the same statistical properties as found in energy transfer in turbulent systems (Deco & Kringelbach, 2020) in a large neuroimaging database (n=1,003) of healthy human participants. Deco and Kringelbach developed the turbulent dynamics framework which we describe in more detail in the **Supplementary Methods**. With a model-free framework this approach captures the unbiased connectivity patterns across the entire connectome of the brain. These patterns have since been shown to be able to distinguish different brain states linked to health and disease (Cruzat et al., 2022; Escrichs et al., 2022) and associate the information cascade, a measure for the transmission of information, as a signature of enhanced information processing (Deco et al., 2021). In addition, Escrichs and colleagues have recently demonstrated that increased levels of whole-brain turbulence before treatment are predictive of responsiveness to pharmacological treatment with escitalopram (Escrichs et al., 2025). Thus, the turbulence-like framework constitutes a potential methodological strategy for identifying informative predictors of antidepressant treatment response, which needs further investigation.

In this work, we investigated whether the turbulence-like fMRI analysis framework can be used to distinguish, before or early during treatment, between patients who will respond and those who will not respond to sertraline treatment in the EMBARC study. We tested three hypotheses: First, that the turbulence framework predicts sertraline treatment outcomes more accurately than chance or standard treatment planning. Second, we hypothesized that increased turbulence and an enhanced information cascade before treatment are predictors of a positive treatment outcome. Third, we hypothesized that this prediction model would be specific to sertraline treatment, i.e., that the model does not generalize to patients initially treated with placebo, but will keep its predictive accuracy in placebo non-responders who are switched to sertraline in second instance.

## 2. METHODS

### 2.1. Participants

Participants were recruited as part of the Establishing Moderators and Biosignatures of Antidepressant Response for Clinical Care for Depression (EMBARC) study (Clinical Trials Registration: NCT01407094), on which details have been reported previously (Trivedi et al., 2016).

Participants were 18–65 years of age with recurrent (at least one prior episode) or chronic (acute episode duration > 2 years) early-onset (before age 30) MDD using the structured clinical interview for DSM-IV diagnosis Axis I Disorders (Spitzer, Williams, Gibbon, & First, 1992). Participants scored ≥ 14 on the 16-item Quick Inventory of Depression Symptomatology (QIDS-SR_16_) (Rush et al., 2003) at both the screening and the baseline visits. Patients were medication-free for at least three weeks before completing any study measures, but were not required to be medication-naive. Exclusion criteria included a history of bipolar disorder or psychosis; substance dependence (excluding nicotine) in the past six months or substance abuse in the past two months; active suicidality; or unstable medical conditions. For the current analyses, patients were excluded for three reasons: 1. for missing HAM-D evaluations at and before the primary endpoint. 2. when they terminated their allocated treatment early. 3. if rs-fMRI data were unavailable or did not meet quality control standards.

Participants provided written informed consent and were recruited from four sites (Columbia University, Massachusetts General Hospital, University of Michigan, and University of Texas Southwestern Medical Center). Institutional review board approval was obtained from each participating site.

### 2.2. Study design

**Figure 1:**
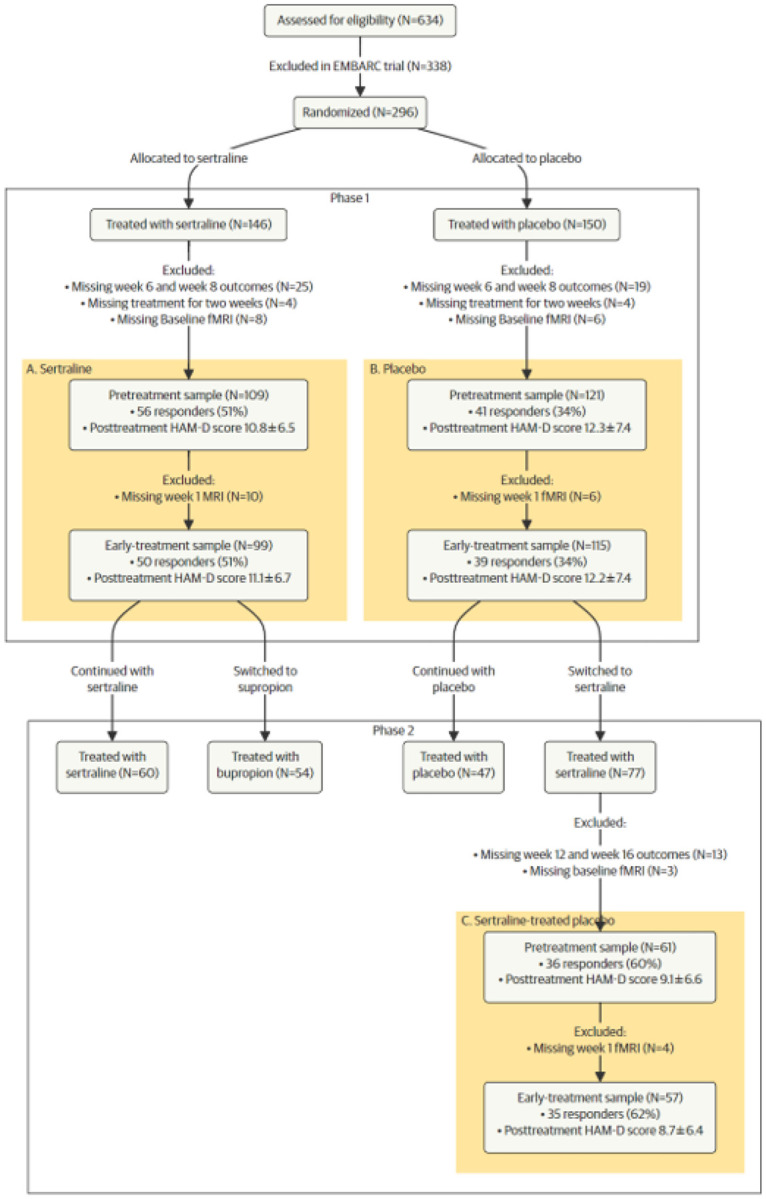
CONSORT Flow Diagram. The EMBARC clinical trial is composed of two study arms and two eight-week stages. The figure highlights the three analysis subpopulations in yellow: A) participants treated with sertraline, B) participants receiving placebo, and C) participants who did not respond to placebo in Stage 1 and were switched to sertraline in Stage 2. For details on the patient selection see the **SupplementaryMethods**.

The EMBARC trial consisted of two eight-week stages (Figure 1). In Stage 1, participants were randomly assigned (1:1) to a blinded eight-week sertraline (Group A) or placebo treatment (Group B), creating two study arms. In Stage 2, participants who did not meet response criteria during Stage 1 were switched, double-blind, to a different treatment for eight weeks. Placebo-non-responders (Group C) were switched to sertraline treatment. A flexible dosing regimen was used, in which medication dosage was increased according to tolerability and response, with the maximum daily dose of sertraline being 200mg. The dosage regimen can be found in theEMBARC study protocol (Trivedi, Fava, Weissman, McGrath, & Parsey, 2012).

To answer our primary research question on the suitability of the turbulent framework for the prediction of sertraline treatment outcome, we analyzed patients assigned to sertraline treatment in Stage 1 (Group A). Thus, this sample was used for training and testing of machine learning models, so-called internal validation. To test the non-generalizability of the model in patients treated in the placebo condition, we tested the machine learning model on the patients assigned to placebo in Stage 1. Thus, this model was trained on sample A and validated externally on the placebo-treated sample (B). To re-test the generalizability of the model in patients treated with sertraline, we tested it on the patients of sample B who did not meet response criteria and were switched to Sertraline treatment in Stage 2 (C).

### 2.3. Primary outcomes

The effectiveness of treatment was assessed using the Hamilton Depression Rating Scale (HAM-D) (Hamilton, 1960). HAM-D scoring was performed at treatment initiation (week 0) and at intervals throughout the 16-week study. The primary endpoint for analysis groups in Stage 1 of the study was week 8 HAM-D score, and week 16 HAM-D score for analysis groups in Stage 2 of the study. In this work, we predict these continuous outcome scores, as well as response, defined as $HAM\;D_{\text{week}8}/HAM\;D_{\text{week}0}<50\%$ (Hamilton, 1960).

### 2.4. MRI acquisition

T1-weighted structural MRI and rs-fMRI data were acquired in the same scanning session before (pretreatment) and again one week after treatment initiation (early-treatment). MRI scan data acquisition has been previously described, and additional pulse sequence information can be found in **Supplementary Table S1**. In short, MRI data were acquired at four sites in the United States, on four different brands of scanners. High resolution, whole-brain, T1-weighted MP-RAGE or equivalent structural scans were acquired (inversion time=900–1100 ms, repetition time=2100–2300 ms, echo time=2.54–3.7 ms, flip angle=9–12°, in-plane matrix=256 × 256 mm, in-plane resolution=1.0 × 1.0 mm, 174 axial slices, slice thickness=0.9 mm). Blood-oxygen level dependent (BOLD) fMRI scans were acquired at rest with the participants staring at a fixed point. fMRI data was captured using a GE-EPI sequence across all machines (repetition time=2000 ms, echo time=28 ms, flip angle=90°, in-plane matrix=64×64 mm, in-plane resolution=3.6 × 3.6 mm, 39 axial slices, slice thickness=3.1 mm, total volumes=180, total scan time was six minutes.

### 2.5. Brain parcellation

A brain parcellation containing 1054 areas was used to extract the average rs-fMRI timeseries signal. This parcellation was a combination of two conventional parcellations. First, in concordance with previous work, we used the publicly available population atlas of cerebral cortical parcellation based on the estimation from a large data set (n=1489) (Schaefer et al., 2018) with 1000 areas. Second, to include the subcortex, we used the 54-area Melbourne Subcortex Atlas (Tian, Margulies, Breakspear, & Zalesky, 2020). All areas were assigned to one of seven RSNs (Yeo et al., 2011) or to the subcortex. For details on the joining procedure of the atlases and definitions of RSNs, see the **Supplementary Methods**.

### 2.6. MRI Preprocessing

MRI data were preprocessed using the fMRIPrep preprocessing pipeline (Esteban et al., 2019) version 20.0.6 for a complete explanation of the fMRIPrep preprocessing pipeline, see the **Supplementary Methods**). Structural T1-weighted MRI was corrected for intensity non-uniformity, normalized, and registered to the Montreal Neurologic Institute (MNI) standard space (matrix = 91 × 109 × 91, resolution = 2 × 2 × 2 mm3). Functional images were corrected for slice-timing, spatially realigned, corrected for spatial distortions using field maps, and co-registered to the structural image, and the corresponding warping map was applied. The normalized functional images were smoothed with a 6 mm full-width half-maximum Gaussian filter. Internal quality control excluded no data based on excessive motion (>2mm) or signal variability (> 4 SDs). Finally, the BOLD time series signals were filtered with a band-pass filter outside the range of 0.008–0.08 Hz to filter low-frequency signal drifts and physiological noise (Cordes et al., 2001; Fox et al., 2005).

### 2.7. Turbulence framework

The turbulence-like approach to analyzing and modeling whole-brain dynamics was first described by Deco and Kringelbach (2020). The interest in using mathematical frameworks originally developed in the modeling of fluid dynamics for the analysis of brain activity is that turbulence facilitates the efficient transfer of energy across scales and that it can be mathematically proven that energy is essentially a form of information (Cross & Hohenberg, 1993; Oono & Yeung, 1987). In their work, Deco & Kringelbach demonstrate that, indeed, information transfer in the brain exhibits the same statistical properties as turbulent energy transfer in fluids.

To model these dynamics, Deco & Kringelbach use a coupled oscillator model (Kuramoto & Kuramoto, 1984) that describes the synchronization between oscillators (i.e., brain areas). The analogy between brain activity and fluid motion can be explained by how synchronization in fluid vortices, whether small or large, resembles the way brain areas synchronize over shorter or longer distances. The scale at which we analyze the turbulent properties of the system is determined by the inverse distance parameter λ, where higher λ values correspond to shorter distances in the brain (λ = [0.01, 0.3] mm^−1^ corresponds to distances ranging from 10 cm to ~3 mm). The synchronization between areas at scale λ is called Kuramoto’s local order parameter *R*_*λ*_. From R, four measures can be computed: Amplitude turbulence, information cascade flow, information cascade, and information transfer, with mathematical definitions provided in the **Supplementary Methods** (Deco et al., 2021).

First, *amplitude turbulence* expresses the variability of synchronization over time and space. It captures *R*_*λ*_ over time, inspired by the rotational vortices found in fluid dynamics.

Second, *information cascade flow* quantifies how synchronized activity at one scale (*R*_*λ*_) predicts information at a slightly finer scale (*R*_*(λ*−*Δλ*)_) at a later time, using a scale step *Δλ* of 0.03 mm^−1^.

Third, information cascade is the average of information cascade flow values across scales, summarizing the brain’s capacity to transfer information consistently across spatial scales.

Finally, *spatial information transfer* indicates how information travels across space at a given scale *λ* by computing the time correlation between *R*_*λ*_ of two brain areas as a function of their distance.

The four measures - and amplitude turbulence for each RSN and subcortex result in 12 features for each of 10 scales between 0.01 and 0.3. Thus, the turbulence framework results in 120 features as input for the pre-treatment model. At early-treatment, the number of features doubles to 240, as all measures are computed for both rs-fMRI scans.

### 2.8. Machine Learning

We trained machine learning pipelines for classifying binary treatment outcome and regression of week 8 HAM-D outcome. Input of the models consisted solely of structured turbulence features as described in the previous paragraph.

The primary performance metrics for classifiers are the area under the receiver operating characteristic (AUROC), the balanced accuracy (bAcc), and the F1-score. For regressors, these were the root mean squared error (RMSE), the mean absolute error (MAE), and the explained variance (R^2^). Here, the error is defined as the difference between the predicted and true week 8 HAM-D score.

We define three analysis samples fitting our research analyses. In our primary analysis, we performed internal validation within analysis sample A using leave-one-out (LOO) cross-validation (CV). For the remaining analyses, we trained on analysis sample A and tested on B, tested on and C. All validations were nested in 5-times repeated 5-fold CV to allow for Bayesian hyperparameter optimization implemented in Scikit-Optimize (v. 0.10.1).

Each machine learning pipeline consisted of three components. First, feature selection which selects the *K* best features based on the univariate linear correlation for regression, or χ^2^-test for classification. The hyperparameter for this pipeline component *K* ranged from 1 to N/5, with N the number of analyzed participants. Second, is scaling, which consists of subtraction of the mean and scaling to unit variance. Finally, a machine learning estimator. For regression we trained linear regression models, and for classification logistic regression classifiers. Both were regularized using elastic net regularization with hyperparameter L1/L2-ratio *α* between 0 and 1. The criterion for classification was accuracy, whilst for regression if was the negative root mean square error (RMSE). Machine learning components were implemented in the Scikit-Learn (v. 1.3.2) (Pedregosa et al., 2011) package for Python (v. 3.9.1).

In addition to performance metrics, we report the most important predictors using impurity-based feature importance. Importance provides a score indicating how useful or valuable each feature was in constructing the boosted decision trees. For more information on the calculation of predictor importance, see the **Supplementary Methods**.

### 2.9. Statistical Analysis

To test whether the performance achieved by our models differs statistically from chance we performed permutation testing implemented in SciPy (v.1.7.3) with 10,000 permutations. The performance metric compared against permutations for classifiers was the AUROC and for regressors the RMSE. P-values were calculated using conservative approximation (Ernst, 2004; Phipson & Smyth, 2010), with a p=0.05 significance threshold. For more details on the calculation of the test statistics see the **Supplementary Methods**.

## 3. RESULTS

### 3.1. Patient Selection

Three hundred nine participants enrolled, of whom 296 underwent at least one baseline assessment. A total of 226 patients were included in the analysis (age 37.9±13.4 years; 148 (65.5%) female; Table 1) with a detailed flow diagram and numbers for each analysis sample shown in Figure 1 (also see the **Supplementary Methods**). In brief, in sample A, 109 participants were included in the pretreatment analysis and 99 in the early-treatment group after exclusions for missing HAM-D scores, early treatment termination, and missing fMRI data. In sample B, 121 participants remained in the pretreatment analysis and 115 in the early-treatment group following similar exclusions. For sample C, 61 participants were included in the pretreatment analysis and 57 in the early-treatment group after accounting for missing outcomes and fMRI data.

### 3.2. Prediction of treatment outcome

**Figure 2:**
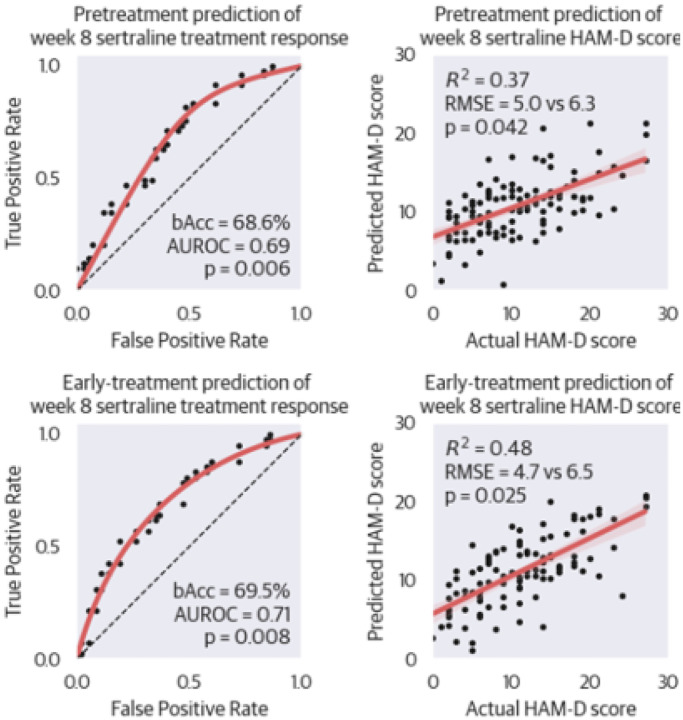
Receiver Operating Characteristics and Regression Plots of Internal Leave-OneOut Validation Performance within the Sertraline Study Arm. The two ROC-curves on the left represent classification performance. The regression plots on the left regression performance. Each dot is the outcome of an individual prediction using LOO validation. Performance is significantly better than chance in all four models. Abbreviations: bAcc=balanced accuracy, AUROC=area under the receiver operating characteristic, HAMD=Hamilton Depression Rating Scale, RMSE=root mean squared error.

First, we evaluated whether it was possible to predict week 8 treatment response based on turbulent measures derived from pretreatment and early-treatment rs-fMRI data. Hyperparameter optimization resulted in an average number of selected features K of 6.9±2.1 features selected and L1/L2-ratio α of 0.44±0.21. Both pretreatment (bAcc=68.6%, AUROC=0.69, p=0.006) and early-treatment (bAcc=69.5%, AUROC=0.71, p=0.008) classifiers were able to perform significantly better than chance. See Table 2 for an overview of all performance metrics and Figure 2 for receiver operating characteristic (ROC) curves.

We also investigated the prediction of week 8 HAM-D symptom severity scores. On average K was 3.7±2.9 features. The models achieved significantly better results than chance using pretreatment (RMSE=5.0, compared to 6.3, p=0.042) and early-treatment (RMSE=4.7, compared to 6.5, p=0.025) data. Regression plots are provided in Figure 2.

**Figure 3:**
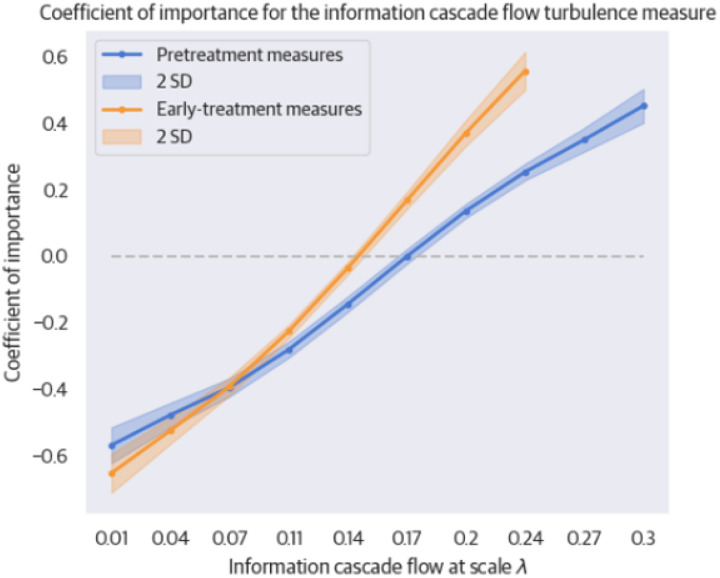
Feature Importance of Information Cascade Flow. The plot shows the coefficient of importance for the information cascade flow turbulence measure from MRI scans acquired pretreatment and one week after treatment initiation (early-treatment). The horizontal axis expresses the scale λ at which the information cascade flow is measured. λ is an inverse distance parameter, such that small values of λ on the left correspond to a larger scale and thus turbulence is measured at greater distances in the brain. The vertical axis shows the coefficient values of feature importance. The magnitude of the value expresses the strength of the feature, whereas the sign expresses its directionality. Thus, high positive values are strongly positively associated with a positive treatment outcome, and low negative values are strongly negatively associated. The magnitude of the coefficient expresses the strength. The shaded regions around each line indicate the variation among models trained in leave-one-out cross-validation, covering two standard deviations. The dashed line at zero serves as a reference point for the coefficients’ significance.

In both the prediction of response and symptom severity, the most selected features were whole-brain information cascade flow measures at different scales. Non-responders to sertraline were characterized by increased long-distance (lower λ’s) and reduced short-distance (higher λ’s) information cascade flow. See Figure 3 for the mean information cascade flow feature importance for different values of λ and **Supplementary Table S2** for the feature importance values.

### 3.3. Sertraline specificity

**Figure 4:**
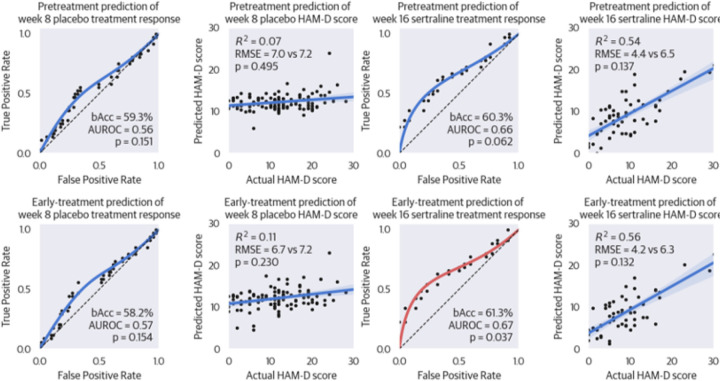
Receiver Operating Characteristics and Regression Plots of Models Tested on the Placebo Study Arm. The four panels on the left display the results for Stage 1 (week 8) of the placebo arm (group B), while the four panels on the right show those for the patients who switched to sertraline in Stage 2 (week 16; group C). Each black dot marks the prediction of an individual patient. Graphs with blue lines did not perform significantly better than chance, whereas predictive performance of the red graph, third in the bottom row is, significantly better than chance. bAcc=balanced accuracy, AUROC=area under the receiver operating characteristic, HAM-D=Hamilton Depression Rating Scale, RMSE=root mean squared eror.

Next, we tested the specificity of machine learning models that were trained on sertraline-treated patients (sample A), to solely predict sertraline response. To this end, we first externally tested the models on all placebo-treated patients (sample B) and then on the non-responders who switched to sertraline in Stage 2 of the study (sample C). In accordance with our hypothesis, all models fared substantially worse when tested the prediction of placebo response in sample B. In sample C, early-treatment prediction of sertraline response performed significantly better than chance (p=0.037), but the other three models failed to do so. See Table 2 for an overview of all performance results and Figure 4 for ROC curves and regression plots of the placebo and sertraline test results.

## 4. DISCUSSION

In this study, we explored whether a turbulence dynamics framework could be used in a machine learning pipeline to predict treatment outcomes for patients with MDD undergoing sertraline treatment. Our results indicate that rs-fMRI features, derived from the turbulence framework, and especially information cascade flow measures, significantly predict antidepressant response, supporting the potential of using turbulence-like whole-brain dynamics for treatment planning in MDD. The findings are particularly relevant given the growing interest in precision psychiatry and the ongoing search for neuroimaging biomarkers to guide treatment decisions in clinical practice.

### 4.1. Prediction of treatment outcome

Our models demonstrated that pretreatment and early-treatment turbulence measures, particularly those related to information cascade flow, can predict both continuous HAM-D scores and binary response outcomes after eight weeks of sertraline treatment. Confirming our first hypothesis, the performance exceeded chance, suggesting that turbulence metrics are valuable predictors of treatment response. Importantly, early-treatment rs-fMRI data further improved the model’s predictive accuracy, implying that brain dynamics captured shortly after treatment initiation may provide additional prognostic information.

These results are consistent with the theoretical foundation that turbulence, characterized by enhanced information transfer and energy dissipation across neural scales, can be indicative of how the brain reacts to antidepressant treatment. Partly confirming our second hypothesis, the feature importance of small- and large scale information cascade flow suggests that increased local synchronization may relate to effective treatment responses. Interestingly, this extends findings from Eschrisch et al., who examined general turbulence properties in a secondary Support Vector Machine (SVM) learning approach as predictors of escitalopram HAMD6 response (Escrichs et al., 2025). As Escrichs et al. (2025) did not report feature importance characteristics, nor showed external replication, this might corroborate that the turbulence framework could be predictive of treatment response, but also suggests that this might be the case across different antidepressants. These findings should further be explored in independent samples when using *a-priori* machine learning to predict treatment outcome. To distinguish the importance of any component of the turbulence framework in a mechanistic pathway in treatment response, placebo-controlled studies with pre-post rs-fMRI measurements are needed.

Compared to more conventional rs-fMRI approaches, the turbulence-based framework demonstrated predictive performance as a single rs-fMRI modality. As demonstrated in previous work (Poirot et al., 2024), static rs-fMRI connectivity analyses, while informative at a population level, appeared to have minimal single-modality predictive features. This limitation is likely due to the reliance on identifying spatially independent networks, which does not fully capture the dynamic and time-varying nature of brain activity. In contrast, turbulence measures reflect the multiscale, dynamic interactions across brain networks, which are crucial for understanding how the brain adapts to pharmacological treatment. This underscores the added value of using complex systems approaches, which might better reflect the pathophysiology of MDD and/or psychiatric disorders in general, and their response to pharmacological intervention. Further application of dynamic analysis techniques across diverse samples would provide valuable insights and strengthen the evidence for their utility in predicting treatment outcomes.

More in general, the performance achieved in this work using the turbulence framework represents a substantial improvement over both conventional rs-fMRI prediction methods and other unimodal approaches to treatment response prediction using MRI (Nguyen, 2019; Nguyen, 2022, Sajjadian, 2023; Poirot, 2024). This suggests that turbulence measures are uniquely sensitive to the complex, dynamic interactions within neural networks that are likely disrupted in MDD. Further research should explore the broader utility of turbulence measures as a single or preferably multi-modality predictor in psychiatric and other complex disorders.

### 4.2. Sertraline specificity

Our third hypothesis, testing the specificity of turbulence measures to drug treatment (i.e., sertraline specificity), was only partially supported. As expected, models trained on sertraline-treated patients performed poorly when applied to placebo-treated patients. However, when applied to patients who switched to sertraline after an initial non-response to placebo (analysis sample C), results were mixed with only replication of the statistical significance of response prediction based on early-treatment effects. Of note, all models still performed better in this group than in the placebo group. This finding is less robust compared to the specificity of our previous multi-modal prediction within the same dataset (Poirot, 2024). Differences could stem from the limited statistical power of the non-parametric test used here and the relatively small sample size of group C. Although we consider this as a strength, sample C patients were selected as non-responders to initial placebo treatment, unlike in sample A; this might potentially have introduced bias complicating our external validation. Finally, treatment response in sample C was defined after 8 weeks of sertraline treatment (but 16 weeks after the initial fMRI-scanning, while in sample A it was assessed after 8 weeks. Therefore, while findings suggest that the predictions based on the turbulence framework could be specific to sertraline, the timing and context of treatment initiation (i.e., direct vs. postponed treatment) may modulate the model’s predictive utility. This warrants further investigation how the timing of rs-fMRI acquisition relative to treatment affects the model’s performance.

### 4.3. Strengths and Limitations

The strengths of this study include the use of the large, well-characterized EMBARC dataset, which allowed for robust testing of the turbulence framework across multiple clinical scenarios. The multi-site acquisition of MRI data, with rigorous preprocessing via fMRIPrep, strengthens the generalizability of our findings, especially in light of the heterogeneity in MRI scanners and protocols across sites. Additionally, our inclusion of both cortical and subcortical regions enhances the comprehensiveness of the turbulence model, as subcortical structures have been implicated in both MDD pathophysiology and antidepressant response.

As a strength, we limited the scope of this work in two ways. First, we did not include clinical predictors in this study to isolate the turbulence framework as a novel method for analyzing rs-fMRI data. However, it is worth noting that incorporating clinical predictors in prior studies has been shown to improve predictive performance (Nguyen, 2020; Sajjadian, 2023; Poirot, 2024). Future work could examine whether combining turbulence measures with clinical data might further enhance prediction accuracy, offering a more comprehensive approach to treatment personalization in MDD. Second, we limited the scope of this work to rs-fMRI, even though a similar performance has previously been demonstrated using task-based fMRI in a very small cohort of 37 subjects (RMSE=4.71) (Nguyen, 2019). The reason we decided to pursue rs-fMRI and not task-based fMRI is that the latter presents several difficulties regarding its scalability and replicability in clinical practice (Klomp 2013).

However, several limitations should be acknowledged. First, as the EMBARC study was conducted within a controlled research environment, the results may not fully translate to routine clinical practice, where treatment adherence and real-world confounding factors (related to less stringent in- and exclusion criteria) may differ (Herzog, 2017; Gomeni, 2023). Therefore, replication of these findings in naturalistic settings is essential to evaluate the clinical utility of turbulence-based predictions. Additionally, because the clinical study was conducted in secondary care, we do not know if these results can be generalized to patients managed by general practitioners, who treat the majority of MDD cases with antidepressants (McManus, 2003).

A second limitation is that this algorithm investigates sertraline response only, while it remains to be tested for other SSRIs and SNRIs also used as first-line drugs in MDD (Simon, 2024). The limitation to a single drug also limits the applicability of the model to predict if a drug is unlikely to result in response. Although this information is valuable, it would be far more valuable to be able to identify the specific antidepressant most likely to achieve a positive response.

Finally, this machine learning algorithm does not necessarily elucidate the underlying neural mechanisms of MDD or treatment response, nor do the predictors found in this work imply any form of causality. Whether prediction will be improved when more mechanistic features are identified and used in machine learning models remains to be shown.

In conclusion, we present a major methodological advancement in the search for resting-state MRI-based neuroimaging biomarkers that -as a single modality- can help to discriminate patients with MDD and select them for treatment optimization. It demonstrates that the turbulence framework can successfully be applied for the prediction of sertraline treatment outcome while the short-distance and increased long-distance information cascade flow was the most important feature to distinguish non-responders. The performance achieved in this work using the turbulence framework demonstrates a substantial improvement over both earlier rs-fMRI-based prediction methods and other unimodal approaches to predicting treatment response using MRI.

#### Supplementary Material

Supplementary Files

This is a list of supplementary files associated with this preprint. Click to download.

- Poirot2024TurbulenceSupplementv3.docx

## Figures and Tables

**Table 1.** Demographic, Clinical, and Outcome Characteristics for Pretreatment Analysis Subgroups.

	A. Sertraline (n=107)		B. Placebo (n=119)		C. Sertraline Treated Placebo (n=61)	
	N	%	N	%	N	%
Female	72	67	76	64	36	59
Race/Ethnicity						
African American	22	21	18	15	10	16
Asian	5	5	9	8	3	5
White	70	65	84	71	44	72
Other	10	9	7	6	4	7
Hispanic	87	81	97	82	51	84
Employed	63	60	69	58	33	54
Responds	56	52	44	37	0	0
	**Mean**	**SD**	**Mean**	**SD**	**Mean**	**SD**
Age (years)	38.6	14	37.2	12.8	38.9	13.2
Body mass index	29.0	8.4	28.0	7.6	26.9	7.3
Years of education	15.2	2.7	15.3	2.4	15.3	2.5
Pretreatment HAM-D score	18.7	4.4	18.5	4.2	18.2	3.9
SHAPS score	33.5	5.4	33	5.9	33	5.4
MASQ score	4.3	3.3	4.3	3.3	4.5	3.2
Change in HAM-D score	−7.8	7.1	−6.4	7.2	−3.1	5.7
Posttreatment HAM-D score	10.9	6.5	12.1	7.5	15.3	5.3

HAM-D=Hamilton Depression Rating Scale, MASQ=Mood and Anxiety Symptom Questionnaire, SHAPS=Snaith-Hamilton Pleasure Scale

**Table 2.** Main Model Performance Metrics for All Analyses.

Classifiers		AUROC	Balanced accuracy (%)	F1-score	p-value
A. Sertraline	Pre-treatment	0.693	68.59	0.586	0.006
	Early-treatment	0.708	69.53	0.557	0.008
B. Placebo	Pre-treatment	0.562	59.33	0.452	0.151
	Early-treatment	0.570	58.24	0.412	0.154
C. Sertraline-treated placebo	Pre-treatment	0.658	60.33	0.733	0.062
	Early-treatment	0.669	61.31	0.753	0.037

Regressors		RMSE	MAE	R^2^	p-value
A. Sertraline	Pre-treatment	5.05	4.22	0.37	0.042
	Early-treatment	4.66	3.78	0.48	0.025
B. Placebo	Pre-treatment	7.00	5.61	0.07	0.495
	Early-treatment	6.73	5.36	0.11	0.230
C. Sertraline-treated placebo	Pre-treatment	4.40	3.38	0.54	0.137
	Early-treatment	4.18	3.46	0.56	0.132

AUROC=area under the receiver operating characteristic curve, bAcc=balanced accuracy, RMSE=root mean squared error, MAE=mean absolute error)

## KEY POINTS

- The turbulence framework produced predictors that were successfully applied for the pretreatment prediction of sertraline treatment outcome in major depressive disorder.
- The specificity of the prediction for sertraline as opposed to placebo treatment was limited.
- Non-responder patients exhibited increased long-distance and reduced short-distance information transfer, as compared to responding patients.
